# Thermoelectric, magnetotransport, and ultrafast dynamics of bismuth telluride thin films grown using pulsed laser deposition: effects of substrate temperature and post-annealing

**DOI:** 10.1080/14686996.2026.2639789

**Published:** 2026-03-19

**Authors:** Le Thi Cam Tuyen, Bih-Show Lou, Jyh-Wei Lee, Ngo Ngoc Uyen, Phuoc Huu Le, Chien-Neng Liao, Chih-Wei Luo, Jiunn-Yuan Lin

**Affiliations:** a Division of Natural Science, Center for Education, Chang Gung University, Taoyuan, Taiwan; b Department of Chemical Engineering and Biotechnology, Tatung University, Taipei City, Taiwan; c Department of Orthopaedic Surgery, New Taipei Municipal TuCheng Hospital, Chang Gung Memorial Hospital, New Taipei City, Taiwan; d Department of Materials Engineering, Ming Chi University of Technology, New Taipei City, Taiwan; e Department of Mathematics–Physics–Informatics, Faculty of Basic Sciences, Can Tho University of Medicine and Pharmacy, Can Tho City, Vietnam; f Center for Plasma and Thin Film Technologies, Ming Chi University of Technology, New Taipei City, Taiwan; g International PhD Program in Plasma and Thin Film Technology, Ming Chi University of Technology, New Taipei City, Taiwan; h Department of Materials Science and Engineering, National Tsing Hua University, Hsinchu, Taiwan; i Department of Electrophysics, National Yang Ming Chiao Tung University, Hsinchu, Taiwan; j Institute of Physics, National Yang Ming Chiao Tung University, Hsinchu, Taiwan; k Faculty of Electrical and Electronics Engineering, Ton Duc Thang University, Ho Chi Minh City, Vietnam; l National Synchrotron Radiation Research Center, Hsinchu, Taiwan

**Keywords:** Bi_2_Te_3_, pulsed laser deposition, thermoelectrics, magnetotransport, pump-probe

## Abstract

n-Type Bi_2_Te_3_ and Bi_4_Te_5_ thin films were grown on SiO_2_/Si substrates via pulsed laser deposition (PLD) at substrate temperatures (*T*_*S*_) ranging from 25°C to 350°C under 220 mTorr He. Film morphology evolved from nanoparticles to layered hexagonal platelets with increasing *T*_*S*_, accompanied by a shift in preferred orientation from (015) to highly (00 l)-oriented textures. Composition varied from Te-rich at low *T*_*S*_ to Te-deficient at 350°C. Near-stoichiometric and (00 l)-textured Bi_2_Te_3_ thin films deposited at 250–300°C exhibited reduced carrier concentration (~9.5 × 10^19^ cm^−3^), significantly enhanced mobility (up to 81.2 cm^2^/V·s), and a maximum thermoelectric (TE) power factor (PF) of 20.0 µW·cm^−1^·K^−2^. To further enhance the TE performance, Bi_2_Te_3_ films grown at 200, 250, and 300°C were in-situ annealed in helium gas at 220 mTorr for 60 min at annealing temperatures (*T*_*A*_) of 200, 250, 300, and 350°C. Simultaneous tuning of *T*_*S*_ and *T*_*A*_ revealed a processing window for optimized PFs, achieving a peak value of 23.8 µW·cm^−1^·K^−2^ for the film grown at 250°C and annealed at 250°C– a 19% improvement over the as-deposited counterpart. Additionally, low-temperature transport measurements exhibited two-dimensional weak antilocalization behavior in the optimized TE Bi_2_Te_3_ thin film, suggesting the presence of topological surface states. Ultrafast spectroscopy further revealed coherent optical and acoustic phonon modes at 1.87 THz and 37.3 GHz, respectively.

## IMPACT STATEMENT

This work systematically optimizes PLD-grown Bi_2_Te_3_ thin films via substrate and annealing temperature control, achieving a PF of 23.8 µW cm^−1^K^−2^, enhanced mobility, and uncovering thermoelectric–topological coupling via magnetotransport and ultrafast spectroscopy.

## GRAPHICAL ABSTRACT

**Figure:**
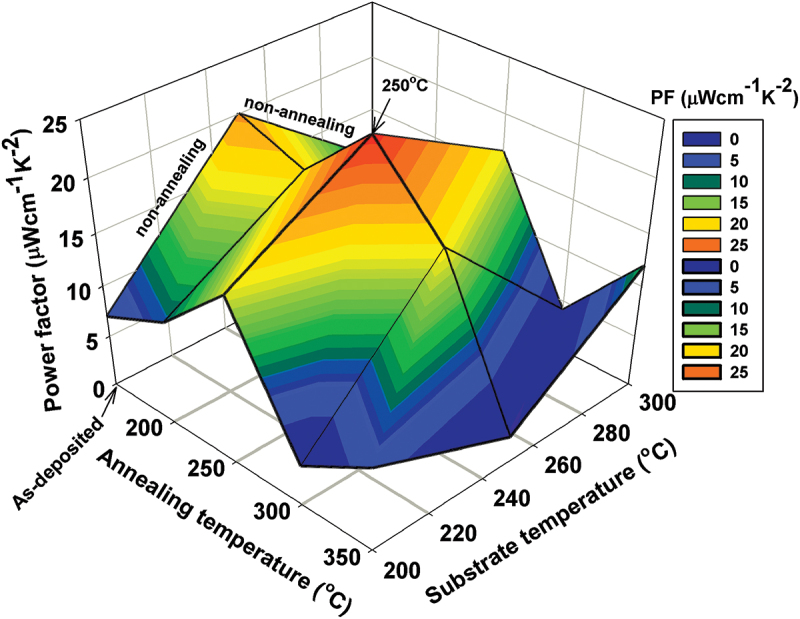

## 1. Introduction

Solid-state thermoelectric (TE) devices provide a direct means to convert waste heat into electricity or function as solid-state coolers, offering advantages such as no moving parts, silent operation, high reliability, and autonomous functionality [1–3]. Growing interest in miniaturized TE systems has driven research toward thin-film devices, which offer superior integration with microelectronics and semiconductor processing [4–6]. Compared to bulk devices, thin-film TEs (thickness ≤10 μm) demonstrate faster thermal response (~15–20 μs vs. 0.35 s for bulk) and can sustain significantly higher heat pumping densities (700–720 W/cm^2^ vs. 10–40 W/cm^2^) [1,6,7].

The performance of TE materials is evaluated by the dimensionless figure of merit, ZT = *σS*^*2*^*T/κ*, where *σ* is electrical conductivity, *S* is the Seebeck coefficient, T is the absolute temperature, and κ is the thermal conductivity. Achieving a high ZT requires maximizing the power factor (PF = *σS*^*2*^) and minimizing *κ*. However, optimizing ZT is challenging due to the coupling between TE parameters [2]. The inverse dependence of *S* on carrier concentration (*n*), as |*S*|~*n*^−2/3^ approximately [2], poses a fundamental challenge to enhancing the PF. Simultaneously, the Wiedemann–Franz law, which links electrical conductivity (*σ*) to electronic thermal conductivity, constrains improvements in the *σ*/*κ* ratio by coupling charge and heat transport. Extensive research efforts to enhance ZT have predominantly focused on two key strategies [8]: (i) reducing the lattice thermal conductivity by incorporating rattler atoms into cage-like structures [9], embedding nanoparticles into the matrix [10], or applying nanostructuring techniques to conventional materials [11,12]; and (ii) improving the power factor (PF) through optimized carrier and band structure engineering [8,13]. This study focuses on the latter strategy, aiming to significantly boost the PF.

Among TE materials, Bi_2_Te_3_ and its alloys remain the most widely used near room temperature owing to their balanced σ, S, and κ values, typically yielding a ZT value close to 1 [8,14–17]. Bi_2_Te_3_-based modules have found applications in automotive waste heat recovery [18], medical cooling devices [19,20], and wearable energy harvesters [21]. For instance, micro-TE coolers based on Bi_2_Te_3_ thin films have been implemented for thermal management in integrated circuits [6], while TEGs have been developed for harvesting body heat [21].

Bi_2_Te_3_-based thin films and their TE properties have been extensively investigated using various fabrication techniques, including sputtering [22–24], thermal evaporation [4], low pressure chemical vapour deposition [25], spin-coating with co-reduction [26], and pulsed laser deposition (PLD) [27–30]. Among these techniques, pulsed laser deposition (PLD) offers distinct advantages for Bi_2_Te_3_ thin film synthesis: (i) stoichiometric transfer from target to substrate via high instantaneous ablation rates, enabling precise compositional control [31]; (ii) high kinetic energy of ablated species (10–100 eV) promoting dense, highly crystalline films with reduced porosity [32]; (iii) independent control of substrate temperature and deposition pressure for systematic growth-parameter optimization [33]; and (iv) compatibility with diverse processing atmospheres and substrate materials. Despite its limitation to smaller substrate sizes (cm-scale), PLD’s capability to produce high-quality, highly textured films makes it well-suited for fundamental thermoelectric property studies [30,34].

A key advantage of TE thin films lies in their fine granular microstructure, which enhances phonon scattering at grain boundaries, thereby reducing lattice thermal conductivity and improving ZT [35]. Among growth parameters, the substrate temperature (*T*_*S*_) plays a critical role in controlling microstructure, crystallinity, and defect density, directly influencing the PF [36]. Post-deposition annealing further enhances TE performance by increasing grain size, reducing defect levels, and improving carrier mobility, which collectively boost σ and PF while minimizing κ [37,38]. Morgan et al. [14] reported a PF of 2.2 μW.cm^−1^K^−2^ in Bi-Te thin films annealed at 300°C on flexible substrates, demonstrating the efficacy of thermal treatment for performance enhancement. However, systematic dual-parameter mapping of the (*T*_*S*_, *T*_*A*_) processing space to identify optimal thermoelectric performance windows remains underexplored in PLD-grown Bi_2_Te_3_ thin films.

Beyond thermoelectrics, Bi_2_Te_3_ is also a well-known 3D topological insulator (TI), possessing conducting surface states protected by time-reversal symmetry [39,40]. The topological surface states (TSSs) in TIs are of interest for spintronic and quantum computing applications [39,40]. Notably, the electronic structure underlying TSSs (characterized by Dirac-cone dispersion and high surface carrier mobility) is intimately connected to the thermoelectric transport properties, as both depend on the material’s band structure, defect density, and carrier scattering mechanisms [41].

TSSs can be probed via 2D weak anti-localization (WAL) behavior in magnetotransport studies [42–44]. However, Bi_2_Se_3_ and Bi_2_Te_3_ are typically heavily doped as a result of vacancies and anti-site defects, leading to dominant bulk conduction that significantly obscures the detection of TSSs [45,46]. Therefore, identifying processing conditions that simultaneously optimize thermoelectric performance (requiring controlled carrier concentration and high mobility) and preserve accessible TSSs (requiring suppression of bulk conduction) represents a critical challenge. Complementary characterization via magnetotransport measurements (probing TSS contribution [42–44]) and ultrafast pump-probe spectroscopy (revealing carrier-phonon coupling and surface-vs-bulk relaxation dynamics [47] – as demonstrated in Bi_2_Se_3_ [46,48], Bi_2_Te_3_ [49,50], and Bi_2_Se_2_Te [51,52]) enables comprehensive evaluation of both quantum transport and dynamical properties in optimized thermoelectric films.

Despite extensive studies on temperature-dependent optimization of Bi_2_Te_3_ thin films, prior PLD investigations have mainly treated substrate temperature (*T*_*S*_) and post-annealing temperature (*T*_*A*_) separately, without systematically mapping the combined (*T*_*S*_, *T*_*A*_) processing space. As a result, simultaneous optimization of thermoelectric performance, topological surface state accessibility, and carrier-phonon dynamics in PLD-grown Bi_2_Te_3_ thin films remains largely unexplored. In this work, n-type Bi_2_Te_3_ thin films were grown by PLD at *T*_*S*_ from room temperature to 350°C, followed by in-situ post-annealing in helium (220 mTorr) at *T*_*A*_ = 200–350°C for 60 min. This dual tuning of *T*_*S*_ and *T*_*A*_ reveals a processing window that maximizes the power factor. The optimized film is then examined by low-temperature magnetotransport to probe TSSs and by ultrafast pump–probe spectroscopy to resolve carrier–phonon dynamics. By correlating processing conditions with thermoelectric, structural, quantum transport, and ultrafast dynamical properties, this study provides guidelines for designing high-performance Bi_2_Te_3_-based thin-film thermoelectrics.

## 2. Experimental details

**Figure 1.**
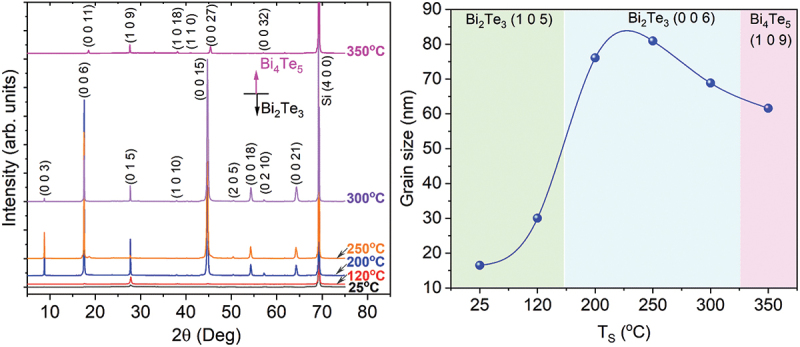
(a) XRD patterns of Bi-Te thin films deposited at various substrate temperature (*T_S_*) ranging from 25 to 350°C. (b) Grain size of the films estimated by Scherer equation.

**Figure 2.**
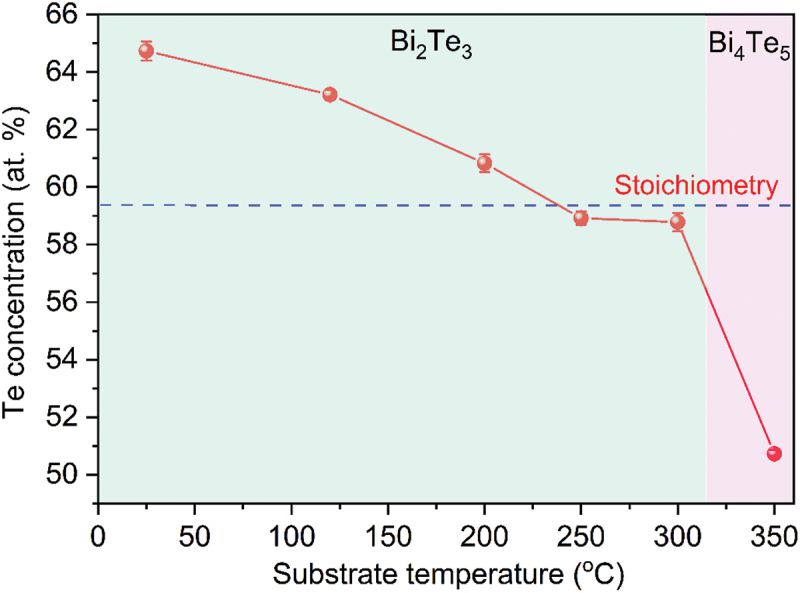
Variation of Te concentration as a function of substrate temperature.

n-Type Bi_2_Te_3_ thin films were fabricated on SiO_2_ (500 nm)/Si (100) substrates (size of 2 × 2 cm^2^) using pulsed laser deposition (PLD) across a substrate temperature (*T*_*S*_) range of 25–350°C. The chamber was evacuated to a base pressure below 3 × 10^−6^ Torr using a mechanical pump followed by a turbomolecular pump. The deposition was conducted in a 220 mTorr helium ambient. Helium was selected as the ambient gas due to its superior thermal conductivity (0.152 vs. 0.018 W/m·K for Ar) enabling uniform substrate heating [53], chemical inertness preventing oxidation, and low atomic mass minimizing re-sputtering damage. The substrate temperature was limited to 350°C to maintain the Bi_2_Te_3_ phase; higher temperatures induce Bi_4_Te_5_ formation via preferential Te re-evaporation (see Figures 1 and 2). A KrF excimer laser (λ = 248 nm) operating at 10 Hz delivered 15–20 ns pulses at a fluence of 3.8 J/cm^2^ per pulse, targeting a stoichiometric polycrystalline Bi_2_Te_3_ source. The target-to-substrate distance was fixed at 40 mm. Each deposition consisted of 15,000 laser pulses over 25 min, resulting in a film growth rate of approximately 0.46 Å per pulse.

Prior to deposition, the chamber was evacuated to a base pressure of 2 × 10^−6^ Torr, followed by helium backfilling and stabilization via differential pumping. To enhance the thermoelectric performance, films deposited at 200, 250, and 300°C were subjected to in-situ post-deposition annealing for 60 min within the PLD chamber under 220 mTorr helium at annealing temperatures (*T*_*A*_) of 200, 250, 300, and 350°C.

The structural properties and crystallographic orientation were analyzed by X-ray diffraction (XRD, Bruker D8, Germany) using Cu Kα radiation (λ = 1.5406 Å) in a 2θ–ω scan mode. Film morphology and thickness were examined by field-emission scanning electron microscopy (FE-SEM, JEOL JSM-6500, Japan) in both top-view and cross-sectional configurations. Elemental compositions were determined via energy-dispersive X-ray spectroscopy (EDS, Oxford Instruments, UK) integrated with the SEM system. Measurements were performed at 15 kV accelerating voltage, with a 90-s acquisition time and dead time between 22% and 30%. Atomic percentages were calculated by averaging data from five randomly selected surface regions.

X-ray photoelectron spectroscopy (XPS, ThermoVG 350, UK) was employed to evaluate the surface composition and chemical states after 5 days of atmospheric exposure. Measurements were carried out using a Mg Kα source (1253.6 eV, 300 W), with calibration to the C1s peak at 284.6 eV. Spectral fitting was conducted using XPSPEAK 4.1 software, employing Gaussian–Lorentzian line shapes and Shirley background subtraction. The in-plane electrical conductivity, carrier concentration, and Hall mobility were measured at room temperature using a van der Pauw configuration (Bio-Rad HL5500PC, USA), with indium contacts ensuring reliable ohmic behavior.

The in-plane Seebeck coefficient was measured at room temperature using a longitudinal DC steady-state method (S = −ΔV/ΔT) [54]. A programmable power supply controlled via LabVIEW established a stable temperature gradient (ΔT = 0.9–1.8 K) across the film, monitored by T-type thermocouples (±0.1 K accuracy). The resulting thermoelectric voltage (ΔV) was recorded using a digital voltmeter with high input impedance (>10 MΩ) to ensure negligible current flow. The Seebeck coefficient was extracted from the linear slope of multiple ΔV vs. ΔT measurements, averaged to ensure reproducibility. Measurement uncertainty was estimated at ±3 µV/K.

Temperature-dependent magnetotransport properties were assessed using a standard four-probe configuration in a physical property measurement system (Quantum Design, USA) from 2 to 10 K. Silver paste was used for electrical contacts. Measurements were conducted with the magnetic field aligned along out-of-plane direction, while both current and thermal gradient were applied parallel to the film plane. Ultrafast carrier and phonon behaviors at room temperature were investigated using a dual-wavelength femtosecond pump–probe technique. A Ti:sapphire laser system, operating at 5.2 MHz with 70 fs pulse duration, provided excitation pulses at 400 nm (3.1 eV) and probing pulses at 800 nm (1.55 eV). During the measurements, the pump and probe fluences were set to 200 µJ/cm^2^ and 9.8 µJ/cm^2^, respectively.

## 3. Results and discussion

### 3.1. Substrate temperature- dependent structural, compositional, electrical, and thermoelectric properties of Bi-Te thin films

Figure 1(a) presents the X-ray diffraction (XRD) patterns of Bi-Te thin films prepared at various *T*_*S*_. For *T*_*S*_ ≤ 300°C, the films exhibited the Bi_2_Te_3_ phase and highly c-axis preferred orientation, characterized by diffraction peaks at 8.8°, 17.5°, 27.7°, 44.7°, 54.4°, and 64.4°, corresponding to the (003), (006), (015), (0015), (0018), and (0021) planes, respectively, consistent with JCPDS card No. 00-015-0863. When *T*_*S*_ was increased to 350°C, the films transition to the Bi_4_Te_5_ phase, displaying sharp and well-defined peaks at 18.5°, 27.5°, 38.4°, 45.5°, and 57.1°, corresponding to the (0011), (109), (1018), (0027) and (0032) planes, respectively. This phase transition at 350°C was attributed to the Te-deficient composition (by 9 *at*.%, Figure 2), due to the sufficient re-evaporation of volatile Te element at the elevated temperature. Additionally, this high temperature increased the ad-atoms mobility, leading to the structural reorganization to form to Bi_4_Te_5_ phase. Researches have shown that higher growth temperatures can induce a transition from the Bi_2_Te_3_ phase to other bismuth-rich phases such as Bi_4_Te_5,_ BiTe, Bi_10_Te_9,_ Bi_4_Te_3_, and Bi_3_Te_3_, as the thermodynamic stability of these phases varies with temperature [55].

As shown in Figure 1(b), the crystallite size distribution was extracted from the XRD data using the Scherrer equation: $=\frac{K\lambda }{\beta \mathrm{cos\theta}}$, where *D* is the average crystallite size, *K* = 0.9 is the shape factor, *λ* = 1.5406 Å is the X-ray wavelength, *β* is the full width at half maximum (FWHM) of the diffraction peak, and *θ* is the Bragg angle. The data reveal a clear temperature-dependent trend: crystallite size increases with *T*_*S*_, peaking at 250°C, and then declines at higher temperatures. At lower *T*_*S*_ (25–120 °C), limited atomic mobility results in smaller crystallites (16.5–30.0 nm). As *T*_*S*_ rises above 120°C, enhanced atomic diffusion facilitates crystallite coalescence, reaching a maximum size of 81.0 nm at 250°C. This behavior is consistent with prior reports, such as Kim et al. [56], who observed similar crystallite growth trends in bismuth telluride thin films with increasing *T*_*S*_. These results align with classical nucleation and growth theories, where thermal activation drives coalescence and grain enlargement [27,30,34]. Beyond 250°C, crystallite size decreases, likely due to increased adatom re-evaporation, or defect generation from thermal stress, which collectively hinder further grain growth [57,58]. Thus, the observed peak at 250°C represents an optimal *T*_*S*_ for promoting crystallite growth, which is critical for tuning the microstructural and functional properties of the thin films.

Figure 2 illustrates the dependence of Te concentration (at.%) on *T*_*S*_, revealing a distinct decline in Te content with increasing *T*_*S*_. At lower *T*_*S*_, the films exhibit a slightly Te-rich composition, with an excess of 4.7 at.% at 25°C and 3.2 at.% at 120°C relative to the stoichiometric ratio (indicated by the dashed blue line in Figure 2). As *T*_*S*_ increases to 200°C, the composition approaches near-stoichiometry (≈60.8 at.%). Beyond this point, a gradual shift toward Te deficiency (by ~1.1–1.2 at.%) is observed at 250–300°C, followed by a sharp decrease to 50.7 at.% at 350°C, where the Bi_4_Te_5_ phase emerges (Figure 1(a)).

This evolution in composition with *T*_*S*_ is governed by the intrinsic differences in vapor pressures and sticking coefficients of Bi and Te. At low *T*_*S*_ (<200°C), both elements exhibit relatively high sticking probabilities [59] and the limited surface diffusion and re-evaporation of ablated species during PLD growth promote retention of the more volatile Te, resulting in a slightly Te-rich film. As the *T*_*S*_ rises (≥250°C), however, the sticking coefficient of Te decreases markedly [59], while its high vapor pressure (P_Te_/P_Bi_ ≈ 10^5^ at 300°C) [27] promotes preferential Te re-evaporation from the heated substrate surface. The combination of reduced Te sticking and accelerated re-evaporation leads to a monotonic suppression of Te incorporation at elevated *T*_*S*_, resulting in a progressive transition from Te-rich to Te-deficient composition as observed experimentally.

**Figure 3.**
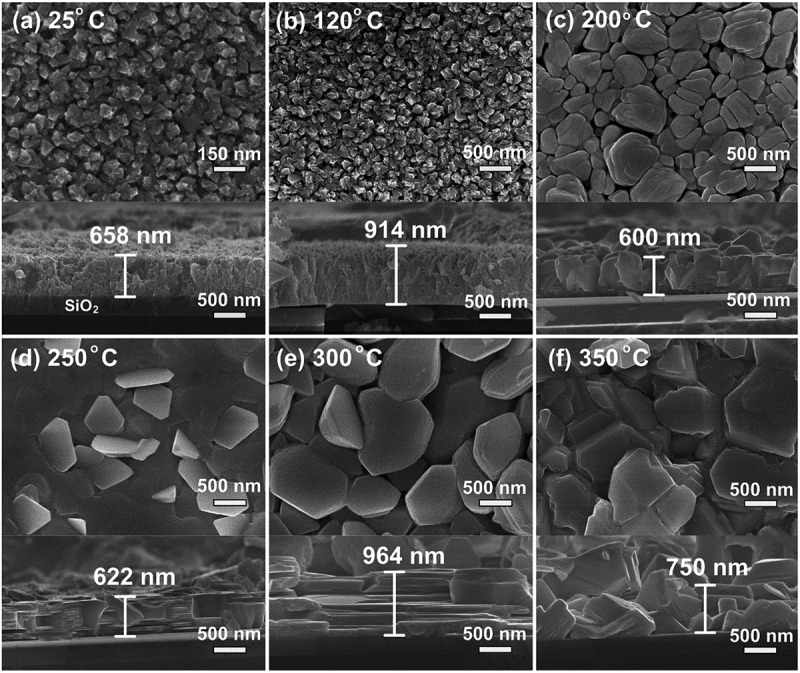
SEM images of the Bi-Te films grown at various substrate temperature (*T_S_*) from 25°C to 350°C.

Figure 3 shows the morphological evolution of Bi-Te thin films grown at different *T*_*S*_ from 25°C to 350°C. The top-view and cross-sectional SEM images provide insights into grain size, surface morphology, and film thickness. At 25°C and 120°C (Figures 3(a,b)), the films exhibit small, densely packed grains of nanoflower-like columnar structures formed with minimal grain coarsening and shadowing growth [60]. As *T*_*S*_ increases to 200°C (Figure 3(c)), the grains become significantly larger, suggesting enhanced atomic mobility and grain coalescence. The grains have rock-like shapes, the film has a thickness of 600 nm and a compact structure (Figure 3(c)). Thin films deposited at 250 and 300°C exhibited distinct layered architectures composed of hexagonal platelets with strong c-axis texturing. This structural preference is likely driven by the anisotropic bonding nature of Bi_2_Te_3_, which promotes much faster crystal growth parallel to the basal planes compared to the c-axis direction [61]. Meanwhile, the layered structure was grown owing to layered structural nature of Bi_2_Te_3_ and high diffusion mobility of adatoms at elevated *T*_*S*_-growth. At 350°C, the film exhibited a rougher surface with larger, polyhedron- and rock-like particles. This morphological change can be attributed to the formation of the Bi_4_Te_5_ phase, whose different crystal structure from Bi_2_Te_3_ disrupts the typical anisotropic growth mode. Moreover, the elevated *T*_*S*_ enhances adatom surface mobility and promotes re-evaporation, leading to island growth, grain coalescence, and surface roughening via step bunching or Ostwald ripening – where larger grains grow at the expense of smaller ones – ultimately resulting in a coarser and less uniform film morphology.

**Figure 4.**
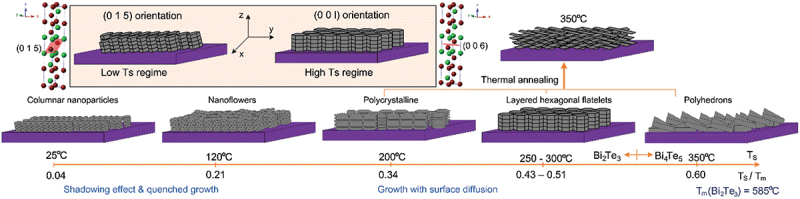
Thin-film growth schematic of Bi-Te thin films at various *T_S_* from 25 to 350°C.

Figure 4 illustrates the growth mechanisms corresponding to the morphological evolution seen in the SEM images. At low substrate temperatures (25–120°C, T_s_/T_m_ = 0.04–0.21), limited adatom mobility and shadowing effects result in a quenched growth regime, forming columnar or rice-like grains with (015) orientation. As *T*_*S*_ increases to 200°C, moderate surface diffusion facilitates polycrystalline growth with improved grain connectivity. In the intermediate *T*_*S*_ range of 250–300°C (T_s_/T_m_ = 0.43–0.51), sufficient adatom mobility and the intrinsically layered structure of Bi_2_Te_3_, combined with its anisotropic bonding, promote the formation of well-aligned hexagonal platelets with strong (00 l) orientation. At 350°C (T_S_/T_m_ = 0.60), the onset of surface and bulk diffusion, coupled with increased Te re-evaporation, disrupts the anisotropic growth mode. This leads to island growth, grain coalescence, and surface roughening, ultimately resulting in the formation of Bi_4_Te_5_ with rock-like and polyhedral morphologies.

**Figure 5.**
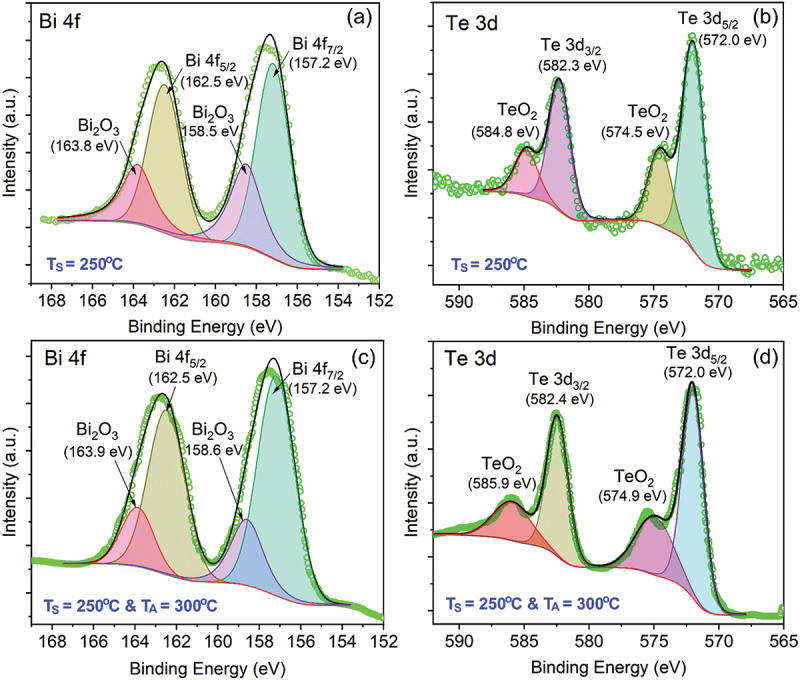
XPS spectra of the Bi 4f and Te 3d core levels for (a, b) the Bi_2_Te_3_ thin film grown at *T_S_* = 250°C, and (c, d) the Bi_2_Te_3_ film grown at *T_S_* = 250°C followed by in-situ thermal annealing at *T_A_* = 300°C for 1 h. All XPS measurements were conducted after five days of exposure to ambient atmosphere. Solid lines denote the fitted curves.

XPS analysis of Bi 4f and Te 3d core levels was conducted to assess the surface composition and oxidation states of the 250°C-Bi_2_Te_3_ thin film after 5 days of air exposure. As shown in Figure 5(a), the Bi 4f spectrum was deconvoluted into two main characteristic peaks of Bi_2_Te_3_, corresponding to Bi 4f_7/2_ at 157.2 eV and Bi 4f_5/2_ at 162.5 eV. In addition, two smaller peaks observed at 158.5 eV and 163.8 eV are attributed to oxidized bismuth species (Bi_2_O_3_). Similarly, in Figure 5(b), the Te 3d region exhibits a clear doublet with peaks at 572.0 eV (Te 3d_5/2_) and 582.3 eV (Te 3d_3/2_), confirming the presence of Te in Bi_2_Te_3_, along with additional TeO_2_ peaks at 574.5 eV and 584.8 eV. The results confirm Bi_2_Te_3_ formation with evident surface oxidation after air exposure. The observed spectral features are in good agreement with previously reported XPS data for Bi_2_Te_3_ single crystals [62], hydrothermally synthesized nanostructures [63], and electrodeposited thin films [64].

To evaluate the effect of *in-situ* thermal annealing during PLD, a Bi_2_Te_3_ film deposited at 250°C and subsequently annealed at *T*_*A*_ = 300°C for 1 h in 220 mTorr helium was examined by XPS. As shown in Figure 5(c,d), the spectrum exhibits dominant Bi 4f and Te 3d peaks of Bi_2_Te_3_, along with evident oxide components – Bi_2_O_3_ (158.6, 163.9 eV) and TeO_2_ (574.9, 585.9 eV) – similar to the non-annealed film (Figures 5(a,b)). Quantitative analysis of the oxide-to-Bi_2_Te_3_ peak area ratios confirms that the Bi_2_O_3_ and TeO_2_ contents are comparable for both films. This indicates that in-situ annealing in a deposition chamber evacuated to high vacuum (<3 × 10^−6^ Torr) and subsequently maintained at 220 mTorr helium does not significantly increase the surface oxidation of Bi_2_Te_3_.

**Figure 6.**
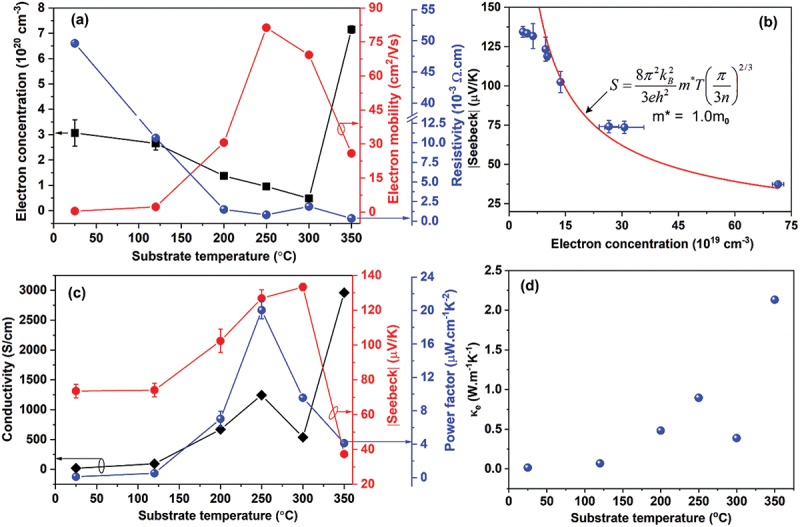
(a) *T_S_* dependence of electron concentrations (*n*), electron mobility (*μ*) and resistivity (*ρ*). (b) Absolute Seebeck coefficient |S| vs. *n*; the solid lines are the plots of the Eq. (1), |*S*|~m*n^−2/3^, m_0_ is the free electron mass. (c) *T_S_* dependence of electrical conductivity (*σ*), Absolute Seebeck coefficient, andpower factor (PF = *S^2^σ*). (d) Electronic part of thermal conductivity for Bi-Te films grown at various *T_S_*.

Figure 6 presents the influence of *T*_*S*_ on the electrical and thermoelectric properties of Bi – Te thin films. As shown in Figure 6(a), the electron concentration (*n*) decreased progressively from 3.1 × 10^20^ to 0.48 × 10^20^ cm^−3^ with increasing *T*_*S*_ from 25°C to 300°C, corresponding to the Bi_2_Te_3_ phase. This reduction is attributed to the suppression of Te-related donor defects, such as antisite Te_Bi_ [65], which has a low formation energy (~0.5 eV) under Te-rich conditions [66,67], and a decrease in Te vacancies as the film composition approaches stoichiometry (see Figure 2) [68]. At 350°C, the carrier concentration (*n*) increased sharply to 7.1 × 10^20^ cm^−3^, which is associated with the formation of a Bi_4_Te_5_ phase with a stoichiometry of 44.4 at.% Bi and 55.6 at.% Te. This composition reflects a notable Te-deficiency of 4.8 at.%, which likely promotes the formation of donor defect of Te vacancies [65,68], thereby enhancing *n*-type conductivity [66,67].

The electron mobility (*µ*) increased markedly from 0.4 cm^2^/V·s at 25°C to a maximum of 81.2 cm^2^/V·s at 250°C, followed by a decline to 69.3 cm^2^/V·s at 300°C and 25.9 cm^2^/V·s at 350°C. This trend is governed by the combined effects of grain size (*D*) and *n*, where *µ* is positively correlated with *D* and inversely related to *n* [65,69]. At low *T*_*S*_, limited grain growth and high defect densities lead to pronounced grain boundary and ionized impurity scattering, resulting in poor carrier mobility [70]. In contrast, films deposited at 250–300°C exhibit larger grains and low *n* associated with near-stoichiometric Bi_2_Te_3_ composition (Figures 1(b) and 2), yielding improved *µ* [70]. The significant drop in *µ* at 350°C is ascribed to enhanced impurity scattering due to the high *n* and the presence of surface roughness and voids (Figure 3). Consequently, the electrical resistivity (ρ = 1/*nµ*e, with e = 1.6 × 10^−19^ C) remained low, ranging from 0.33 to 1.86 mΩ·cm for films grown at *T*_*S*_ between 200 and 350°C (Figure 6(a)), reflecting a favorable balance between carrier concentration and mobility in this temperature window.

The relationship between the Seebeck coefficient (*S*) and carrier concentration (*n*) in metals and degenerate semiconductors (under the parabolic band and energy-independent scattering approximation) is described by [2]:

(1) $$S=\frac{8\pi^{2}k_{B}^{2}}{3eh^{2}}m^{*}T{\left(\frac{\pi }{3n}\right)}^{2/3}$$

where *m*^⁎^ is the carrier effective mass, *T* is temperature, and *n* is the electron concentration. As shown in Figure 6(b), the absolute Seebeck coefficient (|*S*|) exhibits a clear inverse dependence on *n*, decreasing from ~133.4 µV/K at 0.48 × 10^20^ cm^−3^ to 37.3 µV/K at 7.1 × 10^20^ cm^−3^, consistent with the theoretical |*S*| ∝ *m*^⁎^*n*^−2/3^ behavior typical of degenerate semiconductors.

In this work, fitting the experimental data using Eq. (1) (the Pisarenko relation) yielded an effective mass of m* ≈ 1.0 m_0_. In thermoelectric materials, the transport-derived m* is not a fixed band parameter – it depends on valley degeneracy, band anisotropy, and can be significantly elevated by thin-film dimensionality and defect-induced band distortion, while its apparent value also shifts with carrier concentration and the assumed scattering model [71–73]. Correspondingly, a wide range of reported m* values exists for Bi_2_Te_3_ and related materials: m* = 0.19 m_0_ for Bi_2_Te_3_ bulk [74], m* = 0.5 m_0_−0.6 m_0_ for Bi_0.5_Sb_1.5_Te_3_ thin films at 100 K [75], and m* = 0.902 m_0_ for Bi_2_Se_3_ nanosheets synthesized via the polyol method [71]. The effective masses of holes (1.26 m_0_) and electrons (1.07 m_0_) in Bi_2_Te_3_ bulk have also been reported [76], while m* ≈ 1.06 m_0_ for both *n*-type and *p*-type Bi_2_Te_3_ has been reviewed [77]. More recently, m* = 1.0–2.0 m_0_ has been extracted for homo-layer flexible Bi_2_Te_2.85_Se_0.15_ films grown via combined magnetron sputtering and vacuum thermal evaporation [78], which is fully consistent with our fitted value of m* = 1.0 m_0_.

Under Bi-rich growth conditions, Bi_2_ bilayers may intercalate between the quintuple-layered Te^(1)^–Bi–Te^(2)^–Bi–Te^(1)^ structure, forming Bi_2_–Bi_2_Te_3_ superlattice-like films, with carrier concentrations approaching n ~ 10^21^ cm^−3^ [79]. These structural modifications can strongly reshape the band structure, converting the conduction band from single-valley to multivalley, and have been reported to yield markedly higher m* values of 2.78–3.90 m_0_ [79]. Importantly, the moderately enhanced m* = 1.0 m_0_ obtained in this work is likely beneficial, as it increases the density-of-states effective mass without severely degrading mobility, thereby supporting the experimentally observed high Seebeck coefficient even at elevated carrier concentrations (~10^20^ cm^−3^).

A. Novitskii and T. Mori [73] systematically reexamined the Pisarenko relation and demonstrated that, although traditionally associated with the non‑degenerate limit, it remains reasonably accurate into the partially degenerate regime, with its applicability range depending on the dominant scattering mechanism. Comparison with full numerical solutions of the Fermi integrals indicates that the commonly used degenerate approximation may underestimate the density-of-states effective mass at high |S| values (|S| ≳ 150 µV K^−1^), whereas the Pisarenko formulation often provides improved accuracy in this range. Further analysis also emphasizes that the transport-derived effective mass should be interpreted as an apparent parameter reflecting valley degeneracy, band anisotropy, and scattering effects rather than a purely intrinsic band mass. Accordingly, the extracted m* in this work is treated as an effective transport descriptor for comparison with related Bi_2_Te_3_ systems.

Figure 6(c) illustrates the *T*_*S*_- dependent evolution of *σ*, *S*, and PF (= *S*^*2*^*σ*). As *T*_*S*_ increases from 25°C to 250°C, *σ* rises sharply from 20.2 to 1244.7 S/cm, then declines to 538.0 S/cm at 300°C, before peaking at 2960.1 S/cm at 350°C due to phase transition and the highest *n*. Meanwhile, |*S*| shows a non-monotonic trend: it increases from 73.6 µV/K at 25°C to a maximum of 133.4 µV/K at 300°C, then drops to 37.3 µV/K at 350°C (Figure 6c), reflecting the change in *n* and scattering mechanisms.

The highest room temperature PF of 20.0 µW/cm·K^2^ was achieved at *T*_*S*_ = 250°C, where an optimal combination of high conductivity and moderately large Seebeck coefficient (S = −126.8 µV/K) is obtained (Figure 6(c)). Beyond 300°C, the sharp drop in |*S*| outweighs gains in *σ*, leading to a net decrease in PF. These findings highlight 250°C as the optimal deposition temperature for high TE performance in Bi_2_Te_3_ thin films under a helium gas pressure of 220 mTorr.

The electronic thermal conductivity (κ_e_) of the Bi–Te films was estimated using the Wiedemann–Franz law (κ_e_ = σLT, with L = 2.4 × 10^−8^ W Ω K^−2^ for degenerate carriers under elastic scattering). As shown in Figure 6(d), κ_e_ increases markedly with *T*_*S*_, from 0.015 W.m^−1^K^−1^ at 25°C to 2.13 W.m^−1^K^−1^ at 350°C, mirroring the trend in σ (Figure 6(a)). At low *T*_*S*_ (25–120°C), the nanocolumnar ‘nanoflower-like’ microstructure and small grain size (≤30 nm) induce severe carrier scatterings (μ ≤ 2.23 cm^2^/Vs), yielding low σ (≤94.4 S cm^−1^) and thus minimal κ_e_. Increasing *T*_*S*_ to 200–300°C produces well-textured, layered hexagonal platelets with larger grains (68.8–80.9 nm), enhancing carrier mobility (69.3–81.3 cm^2^/Vs) and, consequently, rising κₑ to 0.387–0.896 Wm^−1^K^−1^. At 350°C, the emergence of Bi_4_Te_5_ leads to a very high carrier concentration (*n* ≈ 7.15 × 10^20^ cm^−3^) and reduced μ (25.9 cm^2^/Vs), resulting in a high σ = 2960.1 S cm^−1^ and the peak κ_e_ of 2.13 W.m^−1^K^−1^.

The intermediate κ_e_ values (0.387, 0.483, and 0.896 Wm^−1^K^−1^) obtained for the Bi_2_Te_3_ deposited at T_S_ of 200–300°C are comparable to reported κₑ ≈ 0.4 W m^−1^ K^−1^ for nanocrystalline Bi_2_Te_2.7_Se_0.3_ films [80].

### 3.2. Thermoelectric properties of Bi-Te thin films grown at various substrate temperatures and annealing temperatures

**Figure 7.**
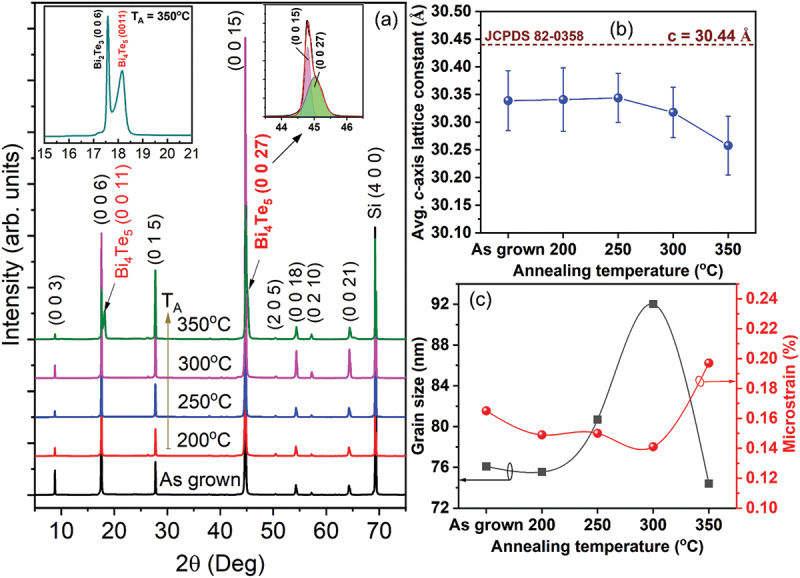
(a) XRD patterns of the Bi_2_Te_3_ thin film grown at 250°C and after *in-situ* thermal annealing at various temperatures (*T_A_*) from 200°C to 350°C. The insets in (a) present zoom-in-view of two highest intensity peaks for the film annealed at 350°C. (b) *T_A_*-dependent *c*-axis lattice constant of the Bi_2_Te_3_ thin films. (c) *T_A_*-dependent grain size and microstrain of the films analysed by Williamson–Hall method.

Figure 7 presents the XRD patterns of the Bi_2_Te_3_ film grown at *T*_*S*_ = 250°C and subsequently subjected to *in-situ* thermal annealing (*T*_*A*_ = 200–350°C). Consistent with the *T*_*S*_-dependent results (Figure 1), all annealed films retain a strong *c*-axis preferred orientation, exhibiting dominant (00 l) reflections along with minor non-(00 l) peaks such as (015), (205), and (0 2 10). For *T*_*A*_ = 200–300°C, only the single-phase Bi_2_Te_3_ was observed. In contrast, annealing at 350°C led to the emergence of a secondary Bi_4_Te_5_ phase, confirmed by the appearance of the Bi_4_Te_5_ (0 0 11) peak at 18.1° and (0 0 27) peak at 45.0° (see insets of Figure 7(a)).

To further assess the effect of thermal annealing on the crystal structure, the average *c*-axis lattice constant was extracted from the (0 0 6) and (0 0 15) reflections using the hexagonal unit cell relation (Eq. 2):

(2) $$\frac{1}{d_{hkl}^{2}}=\frac{4}{3}\left(\frac{h^{2}+\mathrm{hk}+k^{2}}{a^{2}}\right)+\frac{\ell^{2}}{c^{2}}\;\;$$

The *c*-axis lattice constants were 30.34 Å for the as-grown and 200–250°C annealed Bi_2_Te_3_ films, slightly decreasing to 30.32 Å at *T*_*A*_ = 300°C and 30.26 Å at *T*_*A*_ = 350°C. All of these values are lower than the standard lattice constant of 30.44 Å for Bi_2_Te_3_ powder (JCPDS 82–0358), which is commonly attributed to the formation of Te_Bi_ antisite donor defects under Te-rich conditions [81] – due to the smaller atomic radius of Te (1.4 Å) compared to Bi (1.6 Å) – as well as the presence of Te vacancies (V_Te_) under Te-deficient conditions.

The grain size and microstrain of the Bi_2_Te_3_ films were evaluated using the Williamson–Hall method [82], as expressed in Eq. (3):

(3) $$\beta \cos\theta =\frac{\mathrm{K\lambda}}{D_{\beta }}+4\varepsilon \sin\theta \;\;$$

where *β* is the full width at half maximum (FWHM) of the selected diffraction peak at *2θ*, *K* is the Scherrer factor (~0.9), *λ* is the wavelength of the Cu K_α_ radiation (0.15406 nm), *D*_*β*_ is the nanograin size, and *ε* is the microstrain. The grain size (*D*_*β*_) and microstrain (*ε*) are respectively extracted from the intercept and slope of the linear fit in the βcosθ versus 4sinθ plot. The evolution of these parameters with annealing temperature (*T*_*A*_) is presented in Figure 7(c). The microstrain values (0.141–0.197%) indicate that the observed lattice distortion mainly originates from point-defect incorporation rather than macroscopic stress effects. As shown in Figure 7(c), microstrain decreases with increasing *T*_*A*_ from 200 to 300°C, indicating defect relaxation via diffusion at the annealing temperatures. However, it rises considerably to 0.197% at *T*_*A*_ = 350°C, which correlates with the onset of Bi_4_Te_5_ secondary phase formation. Meanwhile, in Figure 7(c), grain size was 76.1 nm for the as-grown film exhibits; it remains unchanged at *T*_*A*_ **=** 200°C (*T*_*A*_ < *T*_*S*_), then increases to 80.7 nm at 250°C and peaks at 92.0 nm at 300°C. At *T*_*A*_ = 350°C, the grain size drops to 74.4 nm, arising from the emergence of secondary Bi_4_Te_5_ phase.

**Figure 8.**
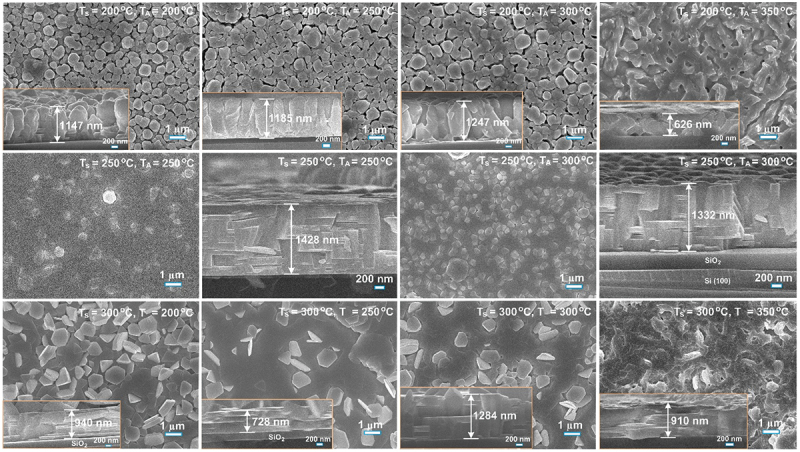
Plan-view and cross-sectional SEM images of Bi_2_Te_3_ films deposited at *T_S_* of 200°C, 250°C, and 300°C, followed by *in-situ* thermal annealing at various temperatures (*T_A_*) from 200°C to 350°C.

To further enhance the TE property, the Bi_2_Te_3_ thin films grown at *T*_*S*_ of 200, 250, and 300°C were subjected to *in-situ* thermal annealing (*T*_*A*_) at 200, 250, 300, and 350°C for 60 min in 220 mTorr helium atmosphere inside the PLD chamber. The *T*_*A*_- dependent morphology change is presented in Figure 8. For the films deposited at *T*_*S*_ = 200°C, a pronounced columnar rock-like morphology is observed. In comparison, the films grown at *T*_*S*_ = 250°C and subsequently annealed at *T*_*A*_ = 250 and 300°C exhibit a mixed surface morphology, characterized by rock-like grains together with poorly defined hexagonal features, accompanied by relatively dense and compact layered structures. Meanwhile, the films deposited at *T*_*S*_ = 300°C show a well-developed layered morphology consisting of hexagonal platelets, indicating enhanced crystalline ordering and anisotropic growth at the higher substrate temperature. These morphologies remain unchanged with annealing at *T*_*A*_ = 200–300°C, though a slight increase in grain size is noted with increasing *T*_*A*_. The increase in grain size with higher *T*_*A*_ is attributed to enhanced atomic mobility, which promotes grain growth and recrystallization [57], reducing grain boundary density, and thus can manipulate carrier transport in the Bi_2_Te_3_ films.

After thermal annealing at 350°C, both Bi_2_Te_3_ films initially grown at *T*_*S*_ = 200°C and 300°C exhibit a marked morphological transformation. The distinct rock-like columnar grains (*T*_*S*_ = 200°C) and layered hexagonal platelets (*T*_*S*_ = 300°C) evolve into a denser, more compact microstructure with indistinct grain boundaries, indicating significant grain merging. This morphological change suggests the onset of partial recrystallization, driven by enhanced atomic diffusion at higher temperatures near 60% of Bi_2_Te_3_‘s melting point (T_m_ ≈585°C). Such thermal energy enables grain boundary migration and coalescence, reducing inter-granular voids and forming a more continuous film. Densification behavior has been observed in Bi_2_Te_3_ films subjected to thermal and laser treatments [83], as well as in hot-pressed Bi_2_Se_0.21_Te_2.79_ bulk materials following thermal annealing and electrical stressing [84].

**Figure 9.**
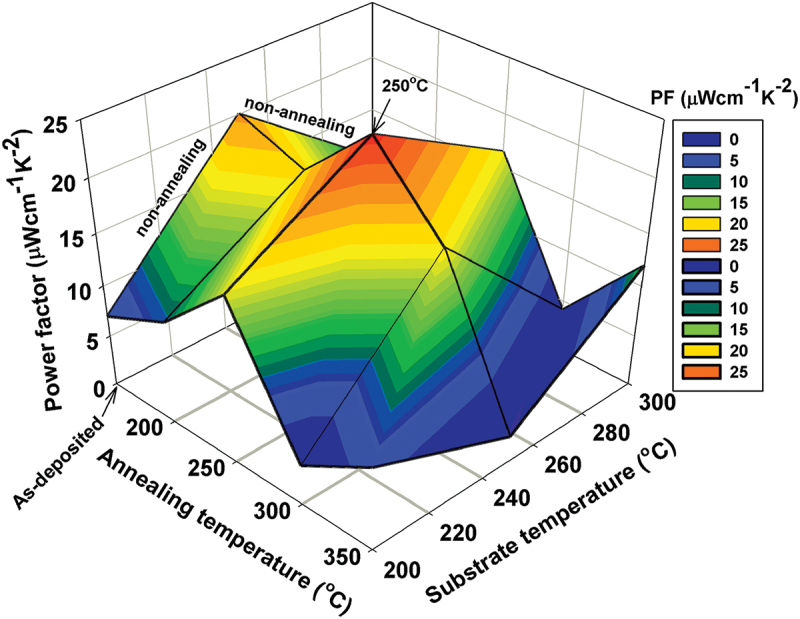
3D contour plot of the film’s power factor (PF = *S^2^σ*) as functions of substrate temperature (*T_S_*) of 200–300°C and annealing temperature (*T_A_*) of 200–350°C.

Figure 9 shows the evolution of PF as a function of *T*_*S*_ and *T*_*A*_, compared to as-deposited films. Thermal annealing notably enhanced PF values in several cases, including films deposited at 200°C and annealed at 200 and 250°C; and those grown at 300°C with the same annealing conditions. Notably, the 250°C-grown-250°C-annealed film exhibited a peak PF of 23.8 µW cm^−1^ K^−2^–representing a 19% increase over the as-deposited counterpart (PF = 20.0 µW cm^−1^ K^−2^). This enhancement is attributed to improved crystallinity, grain growth, and optimized carrier transport due to reduced defect density and better grain boundary connectivity. However, annealing at 300°C and 350°C results in a noticeable decline in the PFs of Bi-Te films compared to their as-prepared counterparts. This suggests that excessive thermal treatment may cause microstructural degradation and phase transformation to Bi_4_Te_5_ that led to the increased *n* and reduced *S*.

Table 1 summarizes the thermoelectric properties of Bi-Te thin films investigated in this study, both before and after annealing, and compares them with relevant results from previous studies. The data clearly show that annealing significantly enhances the thermoelectric performance. For the n-type Bi_2_Te_3_ films prepared in this work, annealing slightly reduces the electrical resistivity (from 0.80 mΩ·cm to 0.79 mΩ·cm) while increasing the absolute value of the Seebeck coefficient (from −126.9 µV/K to −137.9 µV/K), resulting in an improved PF from 20.0 to 23.8 µW·cm^−1^K^−2^.

**Table 1.** Optimized thermoelectric properties of Bi_2_Te_3_-based thin films with and without annealing, compared with previously reported studies.

Material	Method	Conditions	ρ (mΩ⋅cm)	S (µV/K)	Power factor (µWcm^−1^K^−2^)	Ref
n-Bi_2_Te_3_	PLD	As-deposited, *T*_*S*_ = 250°C	0.80	−126.9	20.0	This work
n-Bi_2_Te_3_	PLD	*T*_*S*_ = *T*_*A*_ = 250°C	0.79	−137.9	23.8	This work
n-BiTe	Sputtering	As-deposited, *T*_*S*_ = room temperature	0.77	−32.0	1.33	[14]
n-BiTe	Sputtering	*T*_*A*_ = 300°C, 1 h	0.41	−30.0	2.24	[14]
n-Bi_2_Te_3_	Sputtering	*T*_*S*_ = 350°C	1.97	−102.0	1.49	[23]
n-Bi_2_Te_3_	PLD	*T*_*A*_ = 300°C	—	−188.6	18.1	[85]
n-Bi_2_Te_3_	PLD	*T*_*S*_ = 250°C	0.68	−186.0	50.6	[86]
n-Bi_2_Te_3_	PLD	*T*_*S*_ = 350°C	1.88	−170.0	21.2	[86]
n-Bi_2_Te_3_	PLD	*T*_*S*_ = 300°C	4.35	−91.0	1.9	[28]
n-Bi_2_Te_3_	MBE	*T*_*S*_ = 280°C	1.49	−201.0	27.0	[87]
n-Bi_2_Te_3_	Thermal evaporation	CO_2_ laser annealing at 6.6 mJ/cm^2^	3.5	−165.4	8.2	[83]
CNT/Bi_2_Te_3_	Solution method	*T*_*A*_ = 300°C, 2 h, Ar gas flow	4.2	−175.2	7.4	[37]
n-Bi_2_Te_3_	Co-sputtering	*T*_*S*_ = 225°C	10.5	−55.0	~3.0	[56]
n-Bi_2_Te_3_	Co-sputtering	*T*_*S*_ = room-temperature; *T*_*A*_ = 300°C	0.19	−242	21.0	[88]

Morgan et al. [14] reported pioneering work on n-type Bi-Te thin films fabricated by RF magnetron co-sputtering on flexible polyimide substrates (150 nm thickness), achieving PF = 2.24 µW·cm^−1^·K^−2^ after annealing at 300°C. Their work demonstrated successful implementation on flexible substrates compatible with wearable energy harvesting applications [14]. The significantly lower PF and |S| values (−30.2 µV/K vs. −137.9 µV/K in this work) compared to our rigid-substrate PLD films can be attributed to complementary processing constraints. First, flexible polyimide substrates impose a maximum processing temperature of approximately 300°C due to polymer thermal stability limits, which restricts grain growth and crystallinity enhancement. In contrast, rigid SiO_2_/Si (100) substrates used in this study enable systematic optimization of (*T*_*S*_, *T*_*A*_) up to 350°C. Second, the thinner films in Ref. [14] (150 nm versus ≥600 nm in this work) prioritize mechanical flexibility rather than maximizing power output. Importantly, both approaches are complementary: Morgan’s work establishes scalable and flexible fabrication routes, whereas the present PLD-based study defines performance benchmarks under optimized rigid-substrate conditions. Moreover, PLD and magnetron sputtering offer distinct advantages. PLD typically provides higher plasma kinetic energy, promoting dense and highly textured films with enhanced carrier mobility, albeit limited to centimeter-scale substrates. In contrast, magnetron sputtering supports wafer-scale deposition on both rigid and flexible substrates, making it more suitable for industrial scaling despite generally producing slightly lower-density films.

For PLD-grown Bi_2_Te_3_ thin films, the optimized high PF of 23.8 µW·cm^−1^·K^−2^ for the layered compact-polycrystalline *T*_*S*_=*T*_*A*_ = 250°C-film in this study exceeds the PF reported for nanoparticle-structured Bi_2_Te_3_ films (1.9 µW·cm^−1^·K^−2^) [28] and smooth-polycrystalline films (18.1 µW·cm^− 1^·K^−2^) [85] and is slightly higher than that of compact-smooth films (21.2 µW·cm^−1^·K^−2^) [86]. Although the PF remains below that of smooth epitaxial Bi_2_Te_3_ films grown by molecular beam epitaxy (MBE, 27 µW·cm^−1^·K^−2^) [87], and approximately half of the exceptionally high PF (50.6 µW·cm^−1^·K^−2^) reported for highly (00 l)-oriented layered Bi_2_Te_3_ films deposited at *T*_*S*_ = 250°C [86], it should be noted that those values were achieved under highly controlled epitaxial or strongly texture-engineered growth conditions.

Compared with alternative fabrication approaches, composite films consisting of carbon nanotubes and Bi_2_Te_3_ nanowires subjected to pressing and thermal annealing exhibited a resistivity of 4.2 mΩ·cm, a Seebeck coefficient of −175.2 µV/K, and a PF of 7.4 µW·cm^−1^·K^−2^ [37], which remain substantially lower than the performance achieved in this work. Similarly, optimized Bi_2_Te_3_ films deposited on SiO_2_/Si substrates at 225°C showed a PF of ~3.0 µW·cm^−1^·K^−2^ [56], nearly eight times lower than the present result. Notably, X. Wang et al. [88] reported a relatively high PF of 21.0 µW·cm^−1^·K^−2^ for Bi_2_Te_3_ thin films prepared by RF magnetron co-sputtering at *T*_*S*_ = 150°C followed by post-annealing at 300°C, which is comparable but still slightly lower than the optimized value obtained here. Overall, these comparisons indicate that the PLD-grown Bi_2_Te_3_ thin films developed in this study achieve performance at the upper range of reported polycrystalline systems, approaching epitaxial benchmarks while maintaining practical growth conditions.

For comparison, the total thermal conductivity (κ) in high-performance Bi_2_Te_3_-based systems has been reported as κ = 1.6 Wm^−1^K^−1^ for bulk hot-pressed Bi_2_Te_2.7_Se_0.3_ (ZT ≈ 0.6) [80], 0.61–0.80 Wm^−1^K^−1^ for nanocrystalline Bi_2_Te_3-x_Se_x_ thin films [89], and 0.46–0.81 Wm^−1^K^−1^ for sputtered nanocrystalline Bi – Sb – Te films [90]. For PLD-grown films, κ values range from 0.031 to 0.296 Wm^−1^K^−1^ in Bi_2_Te_3_/Sb_2_Te_3_ superlattice films [91], to approximately 0.8 Wm^−1^K^−1^ (ZT ≈ 0.7) for Bi_2_Te_2.7_Se_0.3_ films [80], and 0.93–1.16 Wm^−1^K^−1^ (ZT = 0.39–0.65 at 300 K) for Bi_x_Sb_2-x_Te_3_ films with grain sizes of 10–190 nm [82]. Considering these reported values for both bulk and thin-film Bi_2_Te_3_-based materials, a representative κ ≈ 1.4 W·m^−1^·K^−1^ can be reasonably assumed for the Bi_2_Te_3_ film (*T*_*S*_ = *T*_*A*_ = 250°C) exhibiting the maximum PF of 23.8 µW·cm^−1^·K^−2^ in this work. Under this assumption, the estimated ZT at 300 K is approximately 0.51, indicating competitive room-temperature thermoelectric performance among PLD-grown polycrystalline Bi_2_Te_3_ thin films.

### 3.3. Magnetotransport property of an optimal thermoelectric Bi_2_Te_3_ thin film

**Figure 10.**
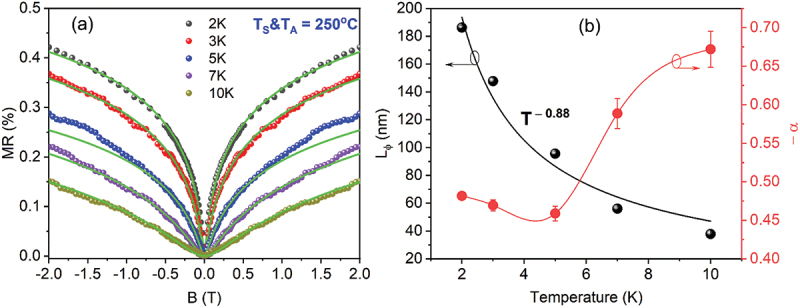
(a) Low-temperature magnetoresistance (MR, ± 2.0 T, 2 – 10 K) curves of the optimized Bi_2_Te_3_ thin film deposited and in-situ annealed at 250°C. The solid green lines at low magnetic fields represent the theoretical fits based on the 2D weak antilocalization (WAL) model using Eq. (4). (b) Temperature dependence of the extracted electron dephasing length *L_ϕ_* and the parameter -*α*. The solid fitting curve shows the power-law dependence of *L_ϕ_(T)* with temperature.

To investigate the signatures of topological surface states (TSSs) in the Bi_2_Te_3_ thin films, temperature-dependent magnetoresistance [MR; MR(%)=[*R*(*B*)−*R*(0)]×100/*R*(0)] measurement was performed on the optimal thermoelectric Bi_2_Te_3_ thin film prepared at *T*_*S*_ = 250°C and *T*_*A*_ = 250°C. Figure 10(a) shows the MR curves recorded between 2 K and 10 K under a perpendicular magnetic field (±2.0T). At low magnetic fields (≤1.0 T), a clear weak anti-localization (WAL) effect is observed, marked by a sharp rise in resistance with increasing field; however, this feature gradually weakens as the temperature increases. In topological insulators (TIs), WAL arises from strong spin–orbit coupling in the bulk and the helical spin texture of surface states [43,46,92,93]. The low-field 2D WAL behavior observed here is well described by the Hikami–Larkin–Nagaoka (HLN) model for systems with strong spin–orbit interaction [46,93], which is

(4) $$\mathrm{\Delta R}(B)[R(0)]2=-\alpha \frac{e^{2}}{2\pi^{2}\hbar }\left[\Psi \left(\frac{1}{2}+\frac{B_{\phi }}{B}\right)-\ln\left(\frac{B_{\phi }}{B}\right)\right]$$

where R denotes the sheet resistance, ΔR=R(B)−R(0), Ψ(x) is the digamma function, $B_{\varphi }=\hbar /(4eL_{\varphi }^{2})$ is the characteristic magnetic field related to the phase coherence length $L_{\varphi }$. The parameter a reflects the number of coherent conduction channels, where *α* = −1/2 corresponds to a single 2D transport channel, and *α* = −1 represents two independent channels with comparable $L_{\varphi }$ values in 3D topological insulators [46,93,94]. The MR results within *B* ±1.0 T fit well with Eq. (4) (green curves in Figure 10(a)), enabling extraction of the temperature-dependent $L_{\varphi }$ and *α* (Figure 10(b)). At 2 K, $L_{\varphi }$ is 186.2 nm – smaller than the reported ~331 nm for a 50-nm-thick Bi_2_Te_3_ film [92] and ~280 nm for Bi_2_Te_3_ microflakes [94]. As temperature increases, *L*_*ϕ*_ steadily declines, following a power-law dependence of *L*_*ϕ*_~*T*^−0.88^. Theoretically, the coherence length follows a power-law dependence of *L*_*ϕ*_*~T*^−0.5^ for dominant e–e scattering in 2D weakly disordered systems, while in 3D systems, with dominant electron–electron and electron–phonon scatterings, it scales as *L*_*ϕ*_*~T*^−0.75^. The observed *L*_*ϕ*_~*T*^−0.88^ suggests the coexistence of TSSs and dominant bulk states with strong dephasing effects.

Figure 10(b) shows the temperature-dependent trend of –α(T), where –α remains in the range of 0.45–0.48 at low temperatures (2–5 K), then increases to 0.59 at 7 K and 0.67 at 10 K. This trend indicates that the film exhibits a single coherent transport channel in the low-temperature regime, while the charge transport channels gradually decouple with increasing temperature. This decoupling occurs because thermal energy disrupts phase coherence, leading to the separation of previously entangled transport pathway. The observation of 2D WAL behavior suggests the presence of entangled phase-coherent channels involving both a 2D TSS and a 3D bulk state in the studied Bi_2_Te_3_ thin films.

### 3.4. Ultrafast dynamics in the optimized thermoelectric Bi_2_Te_3_ thin film

**Figure 11.**
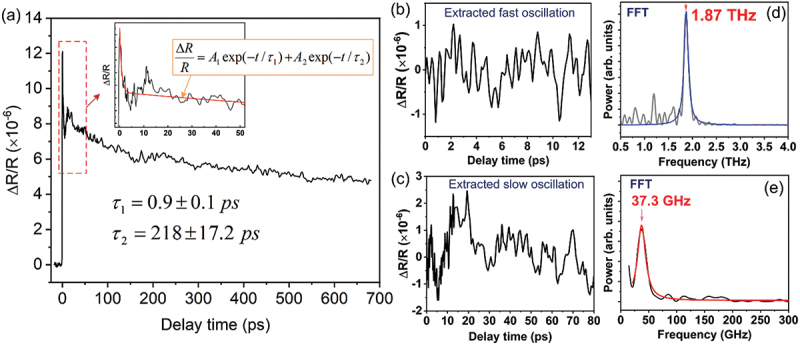
Room-temperature transient reflectivity change (Δ*R/R*) spectrum of the optimized Bi_2_Te_3_ thin film deposited at *T_S_* =250°C and *T_A_* =250°C. The inset shows a magnified view of the signal and its corresponding fit using Eq (5) over the range of −5 ps to 52 ps. Panels (b) and (c) display the extracted fast and slow oscillatory components, respectively. Panels (d) and (e) present the coherent optical and acoustic phonon frequencies, obtained via fast Fourier transform (FFT) analysis of the oscillations in (b) and (c).

Figure 11(a) displays the transient reflectivity change (Δ*R*/*R*) spectrum of the optimized TE Bi_2_Te_3_ thin film, measured at a pump fluence of 0.2 mJ/cm^2^. A sharp initial rise in the Δ*R*/*R* signal reflects the rapid excitation of electrons from the valence band to the conduction band induced by the ultrafast laser pulse. The observed dynamics in Figure. 11(a) were analyzed by fitting the data using Eq (5).

(5) $$\frac{\mathrm{\Delta R}}{R}=A_{1}\exp(-t/\tau_{1})+A{\;}_{2}^{\;}e\mathrm{xp}(-t/\tau_{2})$$

where *A*_*1*_, *A*_*2*_ and *τ*_*1*,_ *τ*_*2*_ are the amplitudes and the relaxation times of hot carriers via various processes, respectively.

The fitting of the *ΔR/R* spectra, as shown in the inset of Figure 11(a), yielded two characteristic relaxation times: τ_1_ = 0.9 ± 0.1 ps and τ_2_ = 218 ± 17.2 ps. The faster component, τ_1_ , corresponds to the relaxation of hot carriers, predominantly governed by electron–electron scattering and electron–phonon interactions. This timescale is consistent with previously reported values for bismuth chalcogenide systems, such as ~1.0 ps in Bi_1.5_Sb_0.5_Te_1.8_Se_1.2_ single crystals [95], ~2 ps in Bi_2-x_Sb_x_Se_3_ [96], and ~2.3 ps in Bi_2_Se_3_ single crystals [97]. The slower relaxation time, τ_2_, likely arises from photocarrier recombination processes and aligns reasonably with the reported value of 111 ps for Bi_2_Te_3_ thin films deposited by PLD [50].

By subtracting the fitted curve from the pump–probe reflectivity spectrum, both fast and slow oscillatory components were isolated (Figure 11(b,c)). These signals were then analyzed using the Fast Fourier Transform (FFT) to identify the corresponding phonon frequencies. A prominent coherent phonon mode was detected at 1.87 THz (Figure 11(d)), consistent with the reported 1.84–1.85 THz longitudinal optical phonon mode in Bi_2_Te_3_ thin films [50,98], which is attributed to the excitation of the $A_{1g}^{1}$ longitudinal optical phonon mode in Bi_2_Te_3_ [99]. Additionally, a second frequency component at 37.3 GHz was observed (Figure 11(e)), which can be reasonably attributed to a bulk acoustic phonon mode of Bi_2_Te_3_. This value aligns with previous findings in Bi_2_Se_2_Te thin films (~33.0 GHz) [51,52] and closely matches acoustic phonon frequencies in other semiconducting materials, such as (001) ZnTe (41 GHz) [100], (100) GaAs (44 GHz) [101], and SnS (39.06 GHz along the zigzag direction) [102].

## 4. Conclusion

This work presents a comprehensive investigation into the fabrication and TE optimization of n-type Bi_2_Te_3_ thin films grown by PLD on SiO_2_/Si substrates. By tuning the *T*_*S*_ from 25°C to 350°C under 220 mTorr helium, we demonstrated clear correlations between growth conditions, morphology, crystal orientation, composition, and TE properties. The film morphology evolved from nanoparticles to layered hexagonal platelets, while the preferred orientation shifted from (015) to highly (00 l)-oriented textures. Near-stoichiometric Bi_2_Te_3_ films formed at 250–300°C exhibited reduced carrier concentration and significantly improved mobility (up to 81.2 cm^2^/V·s), resulting in an enhanced PF of 20.0 µW·cm^−1^·K^−2^. To further boost performance, in-situ annealing at *T*_*A*_ of 200–350°C was applied to films grown at 200, 250, and 300°C. This dual tuning of *T*_*S*_ and *T*_*A*_ revealed a processing window for the optimal TE power factor, achieving a peak PF of 23.8 µW·cm^−1^·K^−2^ for the film deposited at 250°C and annealed at 250°C–a 19% enhancement. Additionally, low-temperature magnetotransport measurements on the optimized TE film revealed two-dimensional weak antilocalization, indicating potential contributions from topological surface states. Ultrafast pump–probe spectroscopy confirmed coherent phonon dynamics, with an optical phonon at 1.87 THz ($A_{1g}^{1}$ mode) and an acoustic phonon at 37.3 GHz. Overall, this study underscores the importance of precise growth and annealing temperatures in tailoring both TE and quantum transport properties of Bi_2_Te_3_ thin films, offering valuable guidance for the development of high-performance, multifunctional thermoelectric materials.
